# Identification of ubiquitination-related gene classification and a novel ubiquitination-related gene signature for patients with triple-negative breast cancer

**DOI:** 10.3389/fgene.2022.932027

**Published:** 2023-01-06

**Authors:** Kankan Zhao, Yuan Zheng, Wenliang Lu, Bo Chen

**Affiliations:** Department of thyroid and breast surgery, Maternal and Child Health Hospital of Hubei Province, Tongji Medical College, Huazhong University of Science and Technology, Wuhan City, Hubei, China

**Keywords:** TNBC, ubiquitination, immune infiltration, prognostic signature, bioinformatics analysis

## Abstract

**Background:** Ubiquitination-related genes (URGs) are important biomarkers and therapeutic targets in cancer. However, URG prognostic prediction models have not been established in triple-negative breast cancer (TNBC) before. Our study aimed to explore the roles of URGs in TNBC.

**Methods:** The Molecular Taxonomy of Breast Cancer International Consortium (METABRIC) and the Gene Expression Omnibus (GEO) databases were used to identify URG expression patterns in TNBC. Non-negative matrix factorization (NMF) analysis was used to cluster TNBC patients. The least absolute shrinkage and selection operator (LASSO) analysis was used to construct the multi-URG signature in the training set (METABRIC). Next, we evaluated and validated the signature in the test set (GSE58812). Finally, we evaluated the immune-related characteristics to explore the mechanism.

**Results:** We identified four clusters with significantly different immune signatures in TNBC based on URGs. Then, we developed an 11-URG signature with good performance for patients with TNBC. According to the 11-URG signature, TNBC patients can be classified into a high-risk group and a low-risk group with significantly different overall survival. The predictive ability of this 11-URG signature was favorable in the test set. Moreover, we constructed a nomogram comprising the risk score and clinicopathological characteristics with favorable predictive ability. All of the immune cells and immune-related pathways were higher in the low-risk group than in the high-risk group.

**Conclusion:** Our study indicated URGs might interact with the immune phenotype to influence the development of TNBC, which contributes to a further understanding of molecular mechanisms and the development of novel therapeutic targets for TNBC.

## Introduction

Breast cancer ranks first in terms of incidence among all cancers according to statistics from the International Agency for Research on Cancer (IARC) (Siegel et al., 2022). Triple-negative breast cancer (TNBC) is the most malignant and aggressive molecular subtype of breast cancer that lacks the expression of the estrogen receptor (ER), progesterone receptor (PR), and human epidermal growth factor receptor 2 (HER2). Compared to other molecular subtypes of breast cancer, TNBC exhibits highly aggressive biological behavior including early recurrences, distant metastases, and a poor survival rate (Waks and Winer, 2019). As endocrine therapy and anti-HER2-targeted therapy were unsuitable for TNBC, chemotherapy and surgery remain the first-line treatments for TNBC with limited efficacy. Although novel therapies including targeted therapy and immune therapy are implemented in clinical practice and clinical trial design (Keenan and Tolaney, 2020; Vagia et al., 2020; Bianchini et al., 2022; Tarantino et al., 2022), clinical outcomes for TNBC remain unsatisfactory. Therefore, the identification of molecules that contribute to risk stratification and clinical decision-making is critical to improve prognosis of TNBC.

Ubiquitination is one of the most common and important post-translational modifications (PTMs). The ubiquitin–proteasome system is a highly specific, ATP-dependent pathway regulating specific protein degradation in eukaryotes. Ubiquitination is a reversible process that is mediated by three types of enzymes, namely, E1 ubiquitin-activating enzyme, E2 ubiquitin-conjugating enzyme, and E3 ubiquitin ligase (Scheffner et al., 1995). E1 activates ubiquitin and transfers it to its activation site Cys in an ATP-dependent manner. E2 transports ubiquitin to E2 itself by binding E1. E3 recognizes substrate proteins and catalyzes ubiquitin transfer from E2 to the substrate. Proteins labeled with ubiquitin are finally taken to the proteasome for degradation. There are other ubiquitin-like modifications, including small ubiquitin-like modifier (SUMO) modification (SUMOylation), pupylation, and ISGlation (Hochstrasser, 2009). The process can be reversed by using deubiquitinating enzymes (DUBs) to cleave ubiquitin and ubiquitin-like molecules from the substrate. In addition, ubiquitin also has many non-degradative functions (Chen and Sun, 2009). As reported by other studies (Ulrich and Walden, 2010; Berndsen and Wolberger, 2014), ubiquitination plays important roles in many cell signaling pathways and biological processes, such as protein activation and transactivation, DNA replication and repair, cell cycle, chromatin dynamics, transcription signaling transduction, autophagy, and immune response, suggesting they are important biomarkers and therapeutic targets. One study constructed a SUMO-related prognostic classifier based on the expression of SUMO1/2/3 and the disease-free survival of TNBC patients (Lin et al., 2021). However, ubiquitination-related gene (URG) prognostic prediction models have not been established in TNBC before.

In the present study, we used the Molecular Taxonomy of Breast Cancer International Consortium (METABRIC) and Gene Expression Omnibus (GEO) databases to screen prognostic URGs. Based on these prognostic URGs, we identified a novel URG-based molecular classification of TNBC. Moreover, we constructed the 11-URG signature with good performance for patients with TNBC. Our analysis suggests that URGs play important roles in TNBC and are potential prognostic biomarkers and therapeutic targets.

## Materials and methods

### Data collection and processing

The gene expression quantification data (HTSeq-FPKM) and corresponding clinic data of patients with TNBC were retrieved from the METABRIC database (http://www.METABRIC.org/) and the GEO database (http://www.ncbi.nlm.nih.gov/geo/). We excluded patients with a survival time of less than 30 days. The gene expression profiles included 297 TNBC patients in METABRIC and 106 TNBC patients in GSE58812. We combined the METABRIC and GSE58812 and removed the batch effects using the ComBat function in the “sva” package (Johnson et al., 2007). We applied principal component analysis (PCA) to test the batch effects. The URGs were downloaded from the ubiquitin and ubiquitin-like conjugation database (Gao et al., 2013) (UUCD) (http://uucd.biocuckoo.org). We merged the URGs and gene expression profiles of TNBC patients to acquire URG expression in both the METABRIC database and the GSE58812 dataset. As a result, 403 TNBC patients with 525 URG expression data and baseline data were included for subsequent analysis.

### Classification of TNBC based on URGs

The univariate Cox proportional hazard regression analysis was used to explore the association of URGs with TNBC patients’ overall survival (OS) and OS time. Those URGs with *p*-value <.01 were considered prognostic URGs. To identify the value of prognostic URGs, we performed non-negative matrix factorization (NMF) analysis to cluster the 297 METABRIC patients and 106 GSE58812 patients. The clustering number K was set as 2–10. We determined the average profile width of a common member matrix by using the NMF package (Gaujoux and Seoighe, 2010) in R with the minimum member numbers of each subclass set to 10. The optimal number of clusters was determined according to indexes including cophenetic, dispersion, evar, residuals, rss, silhouette, and sparseness. Then, we performed Kaplan–Meier curve and log-rank method analysis to evaluate the survival difference between clusters by applying the survminer package in R language. We also calculated the human leukocyte antigen (HLA) expression of different clusters. Based on the Estimation of Stromal and Immune cells in Malignant Tumors using Expression data (ESTIMATE) algorithm (Yoshihara et al., 2013), the immune score, stromal score, ESTIMATE score, and tumor purity of different clusters were determined. Next, we analyzed the enriched pathways between different clusters by applying gene set variation analysis (GSVA) in R. By using “GSEABase” and GSVA R packages (Hänzelmann et al., 2013), we performed single-sample gene set enrichment analysis (ssGSEA) to quantify the extent of the immune-related infiltration of each sample. From a previous study, we collected the gene sets for the evaluation of immune-related characteristics including different types of human immune cell subtypes and immune-related activities (Charoentong et al., 2017; Ru et al., 2019). The enrichment scores calculated using the ssGSEA algorithm indicated the relative degree of each immune-related characteristic expression in each sample. Finally, we applied microenvironment cell populations-counter (Becht et al., 2016) (MCP-counter) and cell-type identification by estimating relative subsets of RNA transcript (Newman et al., 2015) (CIBERSORT) methods to assess the distribution of immune cell infiltration in different clusters.

### Construction, evaluation, and validation of the URG signature in TNBC

The prognostic URGs were entered into the least absolute shrinkage and selection operator (LASSO) method analysis to identify the prognostic multi-gene signature by using the glmnet (Friedman et al., 2010) package in R. Based on the corresponding coefficients and expression of selected genes, the URG signature was constructed as follows: risk score = (β_1_*Gene_1_Exp + β_2_* Gene_2_Exp + β_3_* Gene_3_Exp + ⋯ + β_n_* Gene_n_Exp). In this formula, β represents the coefficients in the LASSO Cox regression analysis. Then, we calculated the risk score for each TNBC patient and classified TNBC patients into a high-risk group and a low-risk group according to the median risk score. The Kaplan–Meier curve and log-rank method were performed to evaluate the OS difference between the high-risk and low-risk groups. The distribution of risk scores, survival statuses of TNBC patients, and expression profiles of prognostic URG were exhibited using R software. We used a time-dependent receiver operating characteristic (ROC) curve to assess the sensitivity and specificity of the URG signature by calculating the area under the curve (AUC) (Heagerty et al., 2000; Blanche et al., 2013). We applied PCA and t-distributed stochastic neighbor embedding (t-SNE) analysis to explore the distribution of 11 URG expression profiles between the high-risk and low-risk groups.

### Estimation of chemotherapy drug sensitivity in TNBC

We used the GDSC database and the pRRophetic package (Geeleher et al., 2014) to estimate the sensitivity of TNBC patients to chemotherapy drugs. We compared the half-maximal inhibitory concentration (IC50) of chemotherapy drugs between high-risk and low-risk group patients. The IC50 values of drugs were negatively correlated with drug sensitivity.

### Construction and evaluation of the nomogram model in TNBC

To verify the independence of the prognostic value of the URG signature and clinicopathological factors (including age, grade, tumor size, lymph node, Nottingham prognostic index (NPI), cellularity, tumor mutation burden (TMB), menopause status, and breast surgery procedure), we performed univariate and multivariate Cox regression analysis to explore their associations with OS of TNBC patients. Factors with *p*-value <.05 in the univariate Cox regression analysis were selected to construct the nomogram model. We used the concordance index (C-index) of 1,000-sample bootstrap and ROC curve to evaluate the prognostic prediction ability of the nomogram model. We also applied calibration curves to further validate the nomogram model.

### Functional enrichment analysis

In order to reveal the heterogeneity between high-risk and low-risk group patients, we performed gene set enrichment analysis (GSEA). Gene Ontology (GO) items of biological process (BP), cellular component (CC), molecular function (MF), the reactome pathway, and the hallmark gene set were selected as the reference gene sets. The results of GSEA were visualized using the enrichplot package in R language.

### Immune infiltration and tumor immune microenvironment analyses

To further explore the immune phenotype between high-risk and low-risk group patients, we conducted immune infiltration and tumor immune microenvironment analyses. The infiltrating score of 16 immune cells and the activity of 13 immune-related pathways were determined by the ssGSEA function of the “gsva” package in R. Based on the ESTIMATE algorithm, we calculated the tumor purity, stromal score, immune score, and ESTIMATE score between high-risk and low-risk group patients. In addition, we performed Spearman’s analysis to explore the correlation of the risk score with the tumor microenvironment.

### Statistical methods

All statistical analyses were performed by R software (version 3.6.1). The Wilcoxon rank-sum test was used to compare the difference between the two groups, and the Kruskal–Wallis test was performed to compare the difference among the four groups. The Kaplan–Meier curve and log-rank method were performed to evaluate the OS difference between groups. The ROC curves were plotted to assess the sensitivity and specificity of the URG signature and nomogram. The correlation between two sets of quantitative data was estimated by Spearman’s correlation test. A two-tailed *p*-value <.05 was considered statistically significant.

## Results

### Identification of prognostic URGs in TNBC

**FIGURE 1:**
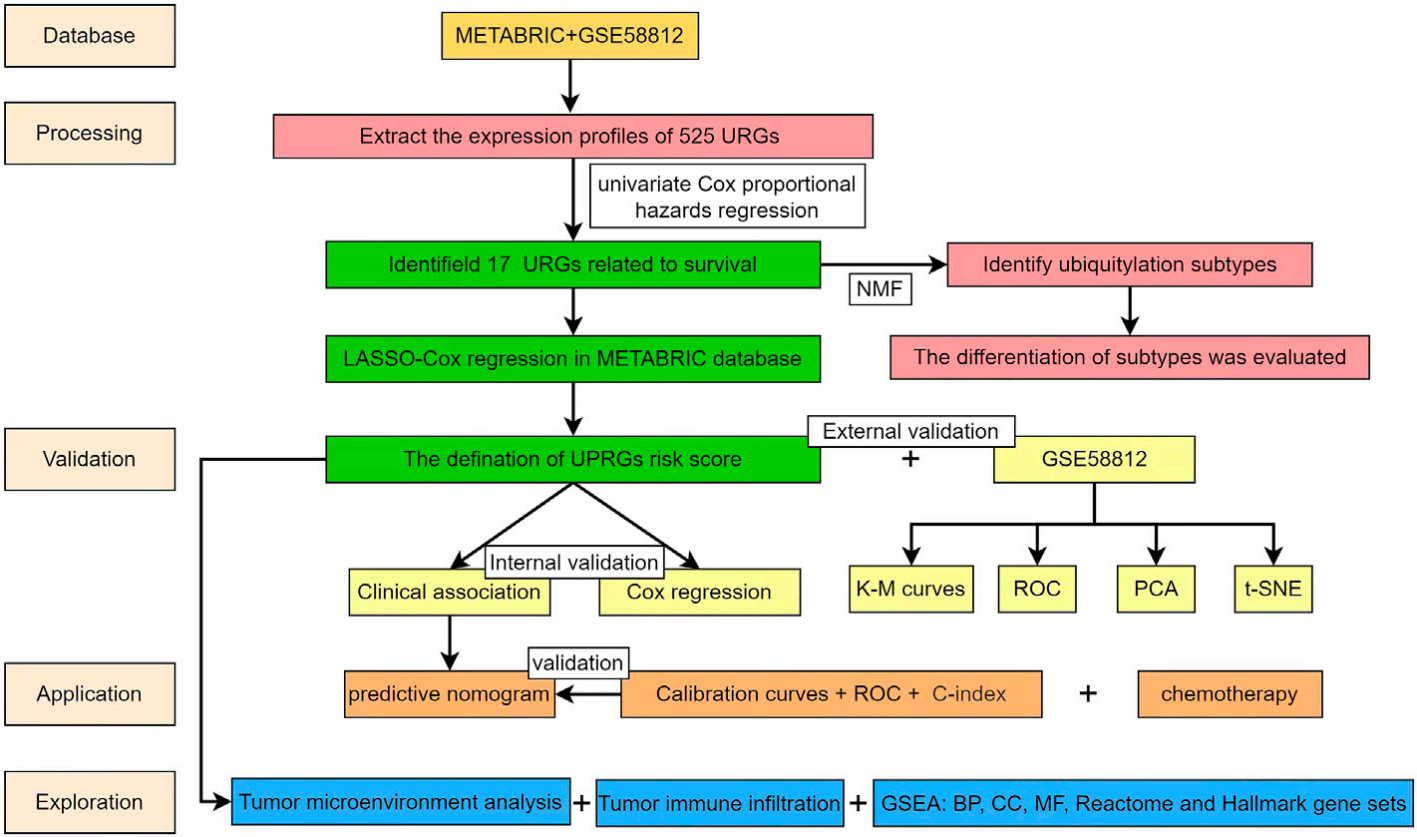
Workflow of the present study.

**FIGURE 2:**
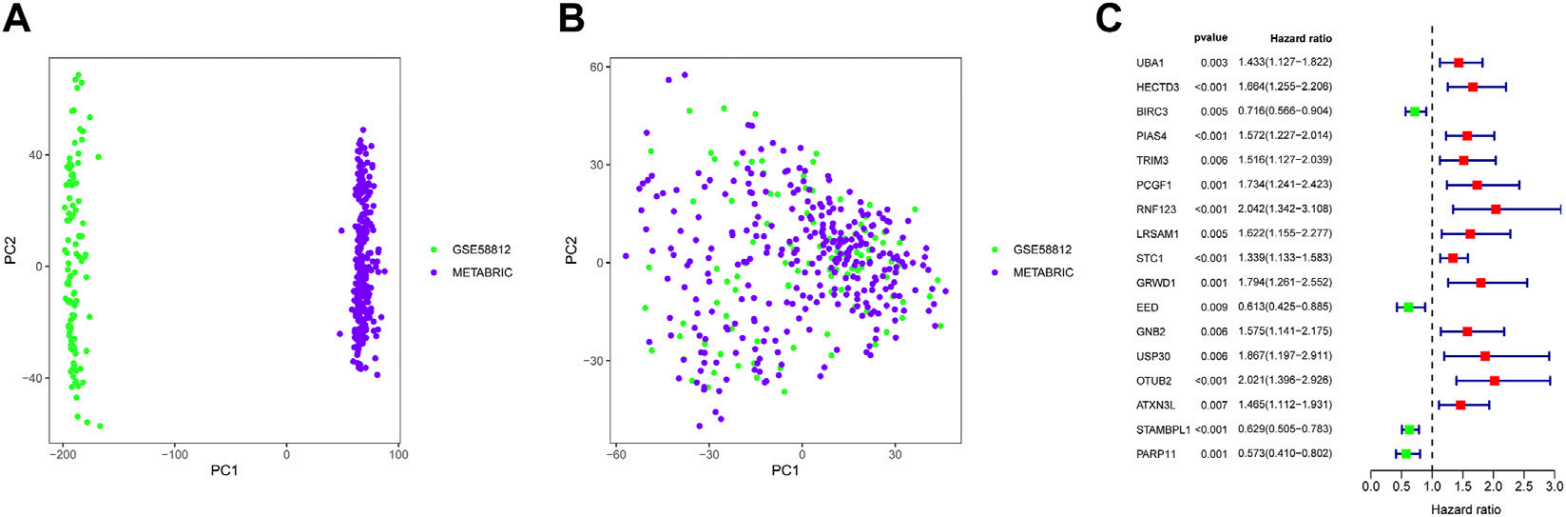
Identification of prognostic URGs in TNBC. **(A)** PCA results before removing batches between the METABRIC database and the GSE58812 dataset. **(B)** PCA results after removing batches between the METABRIC database and the GSE58812 dataset. **(C)** Univariate Cox regression analysis of URGs with OS of TNBC patients. PCA, principal component analysis; OS, overall survival.

The workflow of the present study is presented in Figure 1. Based on the URGs and gene expression profiles of TNBC patients in both the METABRIC database and the GSE58812 dataset, we identified 403 TNBC patients with 525 URG expression data and baseline data. The results of PCA showed that the METABRIC database and the GSE58812 dataset had notable batch effects (Figure 2A), which were removed using the ComBat function in the “sva” package (Figure 2B). These URGs were further assessed for their association with the survival of TNBC. A total of 17 URGs were found to be significantly associated with the OS of TNBC by univariate Cox proportional hazard regression analysis (Figure 2C). Among these 17 URGs, UBA1, PIAS4, TRIM3, PCGF1, RNF123, LRSAM1, STC1, GRWD1, GNB2, USP30, OTUB2, and ATXN3L were found to be the risk factors for TNBC patients [hazard ratio (HR) > 1], and BIRC3, EED, STAMBPL1, and PARP11 were the protective factors of TNBC patients (HR < 1).

### Classification of TNBC based on prognostic URGs

**FIGURE 3:**
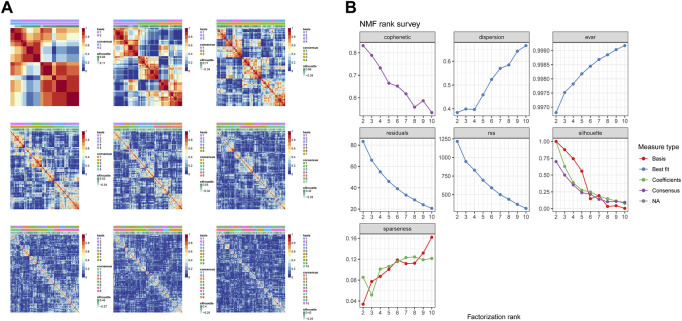
Identification of TNBC subgroups based on prognostic URGs. **(A)** Consensus matrix heatmap with K ranging from 2 to 10. **(B)** Relationship between cophenetic, dispersion, evar, residuals, rss, silhouette, and sparseness coefficients with respect to the number of clusters.

**FIGURE 4:**
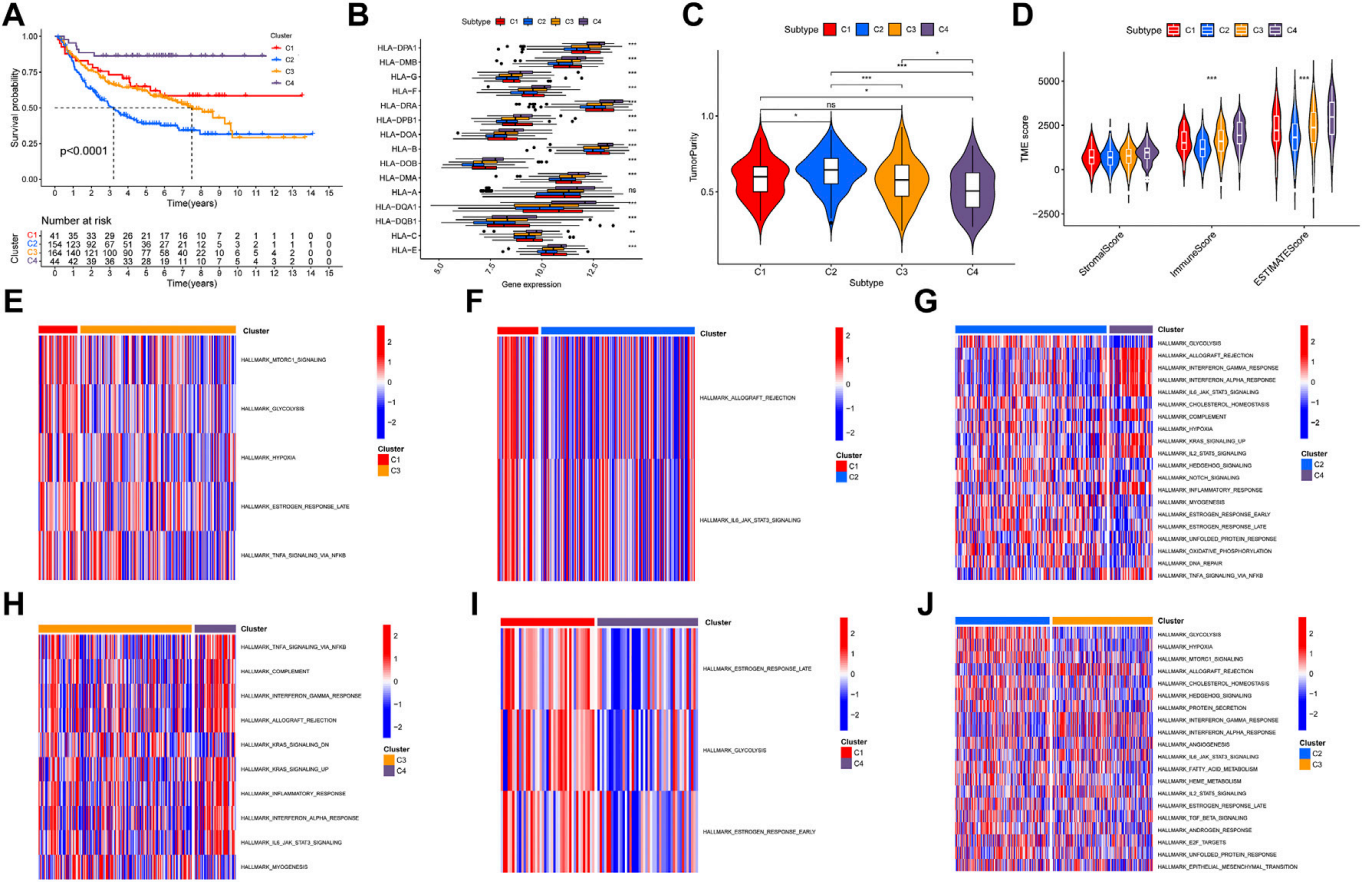
Evaluation of the characteristics of TNBC clusters. **(A)** Kaplan–Meier curve of patients in four TNBC clusters. **(B)** HLA expression in four TNBC clusters. **(C)** Violin plot of tumor purity in four TNBC clusters. **(D)** Violin plot of tumor microenvironment score in four TNBC clusters. **(E–J)** Heatmap of different pathways between four clusters.

**FIGURE 5:**
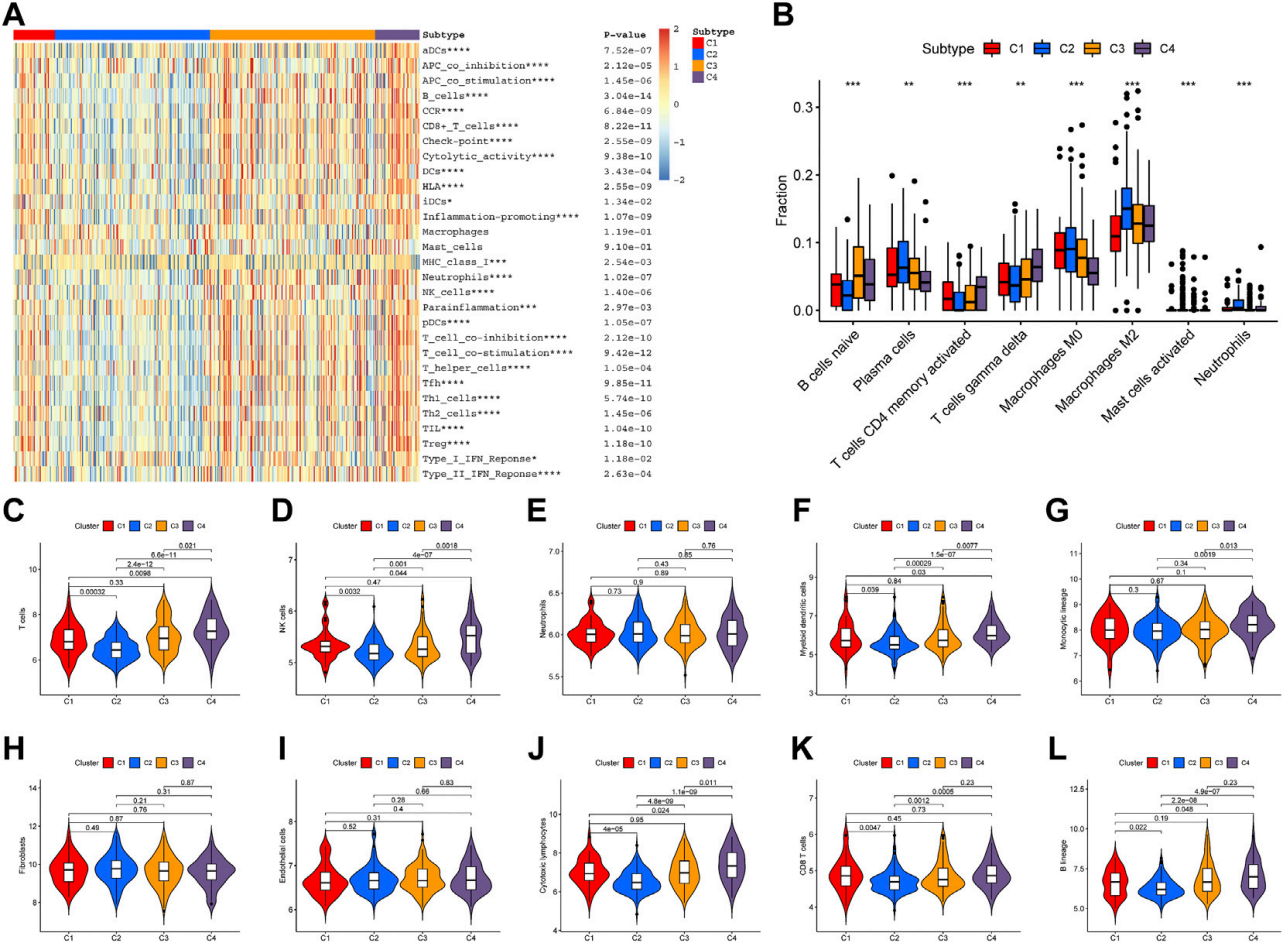
Immune infiltration among four clusters. **(A)** Heatmap of immune cell infiltration and immune-related activities in four clusters. **(B)** CIBESORT analysis of immune cell infiltration among four clusters. **(C–L)** MCP-counter analysis of 12 immune cells’ distribution among four clusters.

A total of 17 prognostic URGs were used as variables of consensus clustering by using the NMF package. Figure 3A depicts the consensus matrix heatmap with K from 2 to 10. According to the cophenetic, dispersion, and silhouette curves (Figure 3B), the optimal number of subgroups was determined as 4 (K = 4). Then, 403 TNBC patients could be classified into four robust clusters, including 41 patients in cluster 1, 154 patients in cluster 2, 164 patients in cluster 3, and 44 patients in cluster 4. The expressions of 17 prognostic URGs among four clusters are significantly different (Supplementary Figure S1). The Kaplan–Meier curve showed that patients in different clusters have significantly different prognoses (*p* < .0001, Figure 4A). Cluster 2 patients had the worst OS, and cluster 4 patients had the best OS among all clusters. To explore the mechanism of survival difference between clusters, we analyzed HLA expression, the immune microenvironment, and pathways between these four clusters. As to HLA expression, 14 of 15 HLA expressions were lowly expressed in cluster 2 and highly expressed in cluster 4. Among these four clusters, cluster 2 patients had higher tumor purity and lower immune and ESTIMATE scores, while cluster 4 patients had lower tumor purity and higher immune and ESTIMATE scores (Figures 4C, D, and Supplementary Figure S2). The enriched pathways between different clusters are shown in Figures 4E–J. Glycolysis, cholesterol homeostasis, hypoxia, NOTCH signaling, and DNA repair were the mainly upregulated pathways in cluster 2, while interferon response, the IL6–JAK-STAT3 signaling pathway, the IL2–STAT5 signaling pathway, the inflammatory response, and the TNF α signaling pathway were the mainly upregulated pathways in cluster 4. The immune cell infiltration and immune-related activities among four clusters are set out in Figure 5 and Supplementary Figure S3. T cells, CD8 T cells, NK cells, myeloid dendritic cells, cytotoxic lymphocytes, and B lineage were significantly downregulated in cluster 2, while M2 macrophage was significantly upregulated in cluster 2. Immune-related activities including cytolytic activity, inflammation-promoting activity, and type II IFN response were inhibited in cluster 2. Cluster 2 exhibited the immune desert phenotype, and cluster 4 exhibited the immune-enriched phenotype, which may account for the OS difference between clusters 2 and 4.

### Construction, evaluation, and validation of the URG signature in TNBC

**FIGURE 6:**
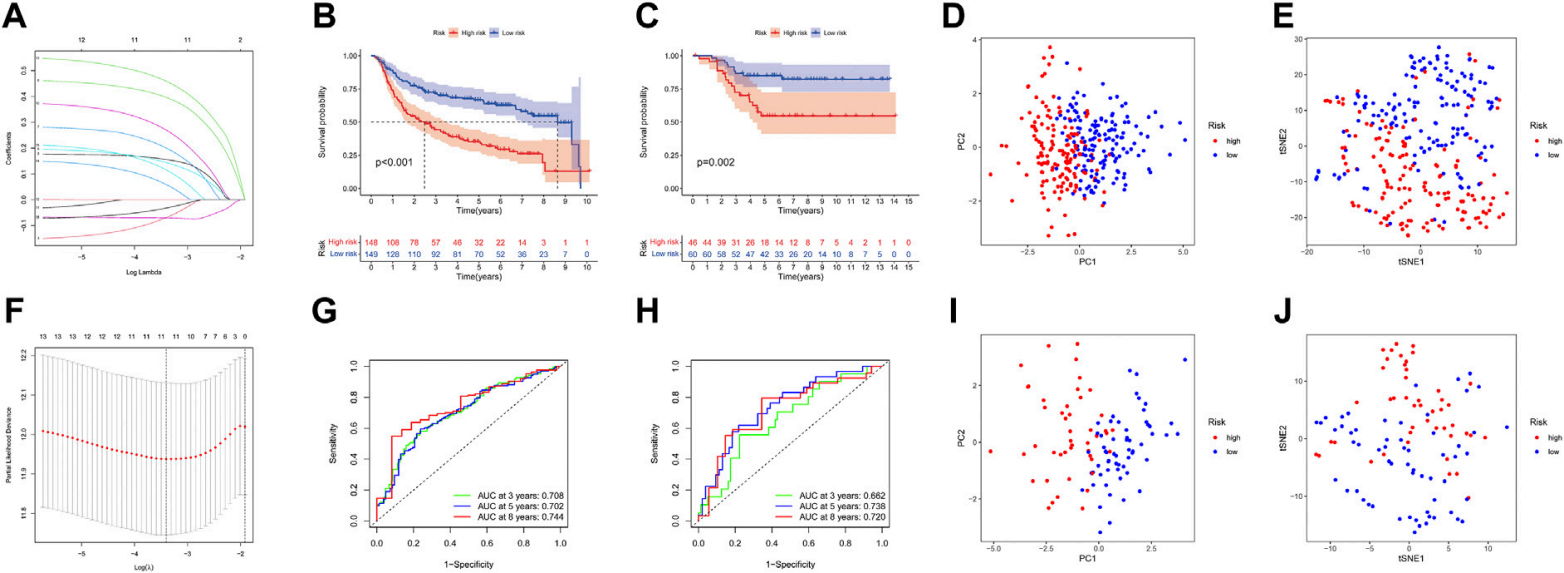
Construction and evaluation of the prognostic URG signature for TNBC patients. **(A)** LASSO coefficient profiles of URG. **(B**,**C)** Kaplan–Meier curve of TNBC patients according to the 11-URG signature in the training and test cohorts. **(D**,**E)** PCA and t-SNE analysis between high-risk and low-risk groups in the training cohort. **(F)** “Leave-one-out cross-validation” for parameter selection in the LASSO model. **(G**,**H)** ROC curve of the 11-URG signature for predicting 3-, 5-, and 8-year OS of TNBC patients in the training cohort and test cohort. **(I,J)** PCA and t-SNE analysis between the high-risk and low-risk groups in the test cohort. LASSO, least absolute shrinkage and selection operator; PCA, principal component analysis; t-SNE, t-distributed stochastic neighbor embedding; ROC, receiver operating characteristic; and OS, overall survival.

**FIGURE 7:**
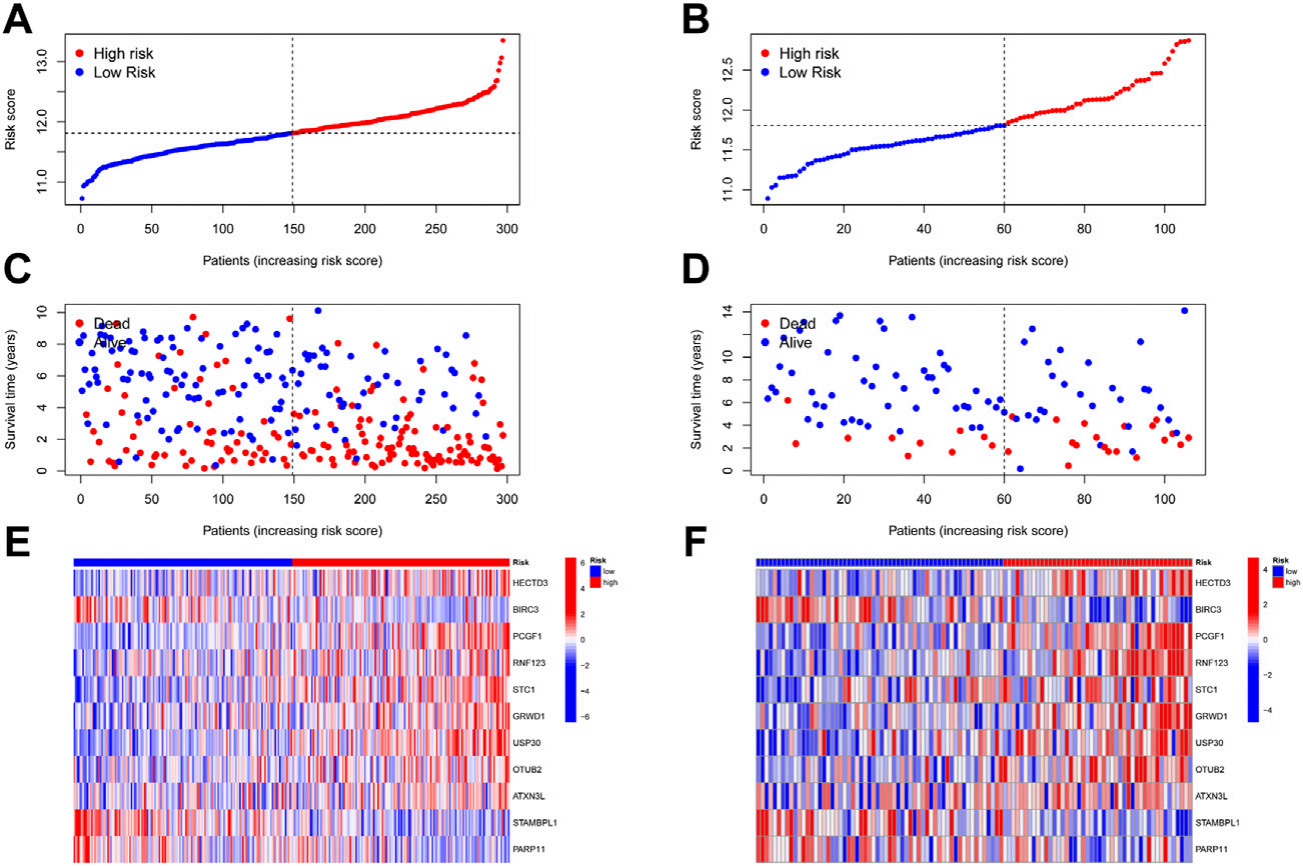
Distribution of risk score, survival status, and expression profile of 11 URGs in the training group and test group. **(A,B)** Distribution of risk score in the training and test cohorts, respectively. **(C,D)** Survival status, survival time, and risk score in the training and test group cohorts, respectively. **(E,F)** Expression profile of 11 URGs between the high-risk and low-risk groups in the training and test cohorts, respectively.

A total of 17 prognostic URGs were fit into the LASSO Cox analysis to identify the optimal prognostic URGs in the training group (METABRIC). We identified 11 URGs (HECTD3, PCGF1, RNF123, STC1, GRWD1, USP30, OTUB2, ATXN3L, BIRC3, STAMBPL1, and PARP11) using LASSO Cox analysis and constructed a prognostic signature by integrating the 11 URG expression profiles and corresponding Cox regression coefficients (Figures 6A, F). We calculated the risk score for each patient in the training group and ranked them into a high-risk group (*n* = 148) and a low-risk group (*n* = 149) according to the median risk score. The Kaplan–Meier curve showed that patients in the high-risk group have significantly worse OS than patients in the low-risk group (*p* < .001, Figure 6B). The prognostic power of the 11-URG signature was evaluated by calculating the AUC. The results showed that the AUC of the 11-URG signature for predicting 3-, 5-, and 8-year survival of TNBC patients was 0.708, 0.702, and 0.744, respectively, which indicated good performance (Figure 6G). PCA and t-SNE analysis showed that TNBC patients between the high-risk and low-risk groups can be distinguished well according to this signature (Figures 6D, E). To verify the reliability of the 11-URG signature in TNBC, we applied this signature to the test set (GSE58812). We calculated the risk score for each patient in the test group and ranked them into a high-risk group (*n* = 46) and a low-risk group (*n* = 60). As presented in Figure 6C, patients in the high-risk group have significantly worse OS than patients in the low-risk group (*p* = .002). The AUC of the 11-URG signature for predicting 3-, 5-, and 8-year survival in the test set was 0.662, 0.738, and 0.720, respectively (Figure 6H). The results of PCA and t-SNE analysis in the test group also showed that patients in the high-risk and low-risk groups were distributed in two directions according to this signature (Figures 6I, J). The distribution of risk score, survival status of TNBC patients, and the expression profiles of 11 prognostic URGs in the training and test sets are displayed in Figure 7. The mortality was much higher for patients with a high risk score than those with a low risk score, and patients in the high-risk group have a tendency toward higher expression of HECTD3, PCGF1, RNF123, STC1, GRWD1, USP30, OTUB2, and ATXN3L and lower expression of BIRC3, STAMBPL1, and PARP11.

### Construction and evaluation of the nomogram model in TNBC

**FIGURE 8:**
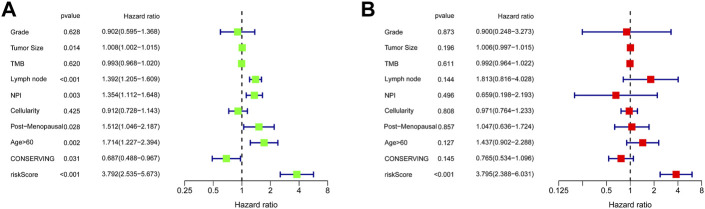
Univariate and multivariate Cox regression analyses of the association of the risk score and clinicopathological factors with the OS of TNBC patients. **(A)** Univariate Cox regression analysis. **(B)** Multivariate Cox regression analysis. OS, overall survival.

**FIGURE 9:**
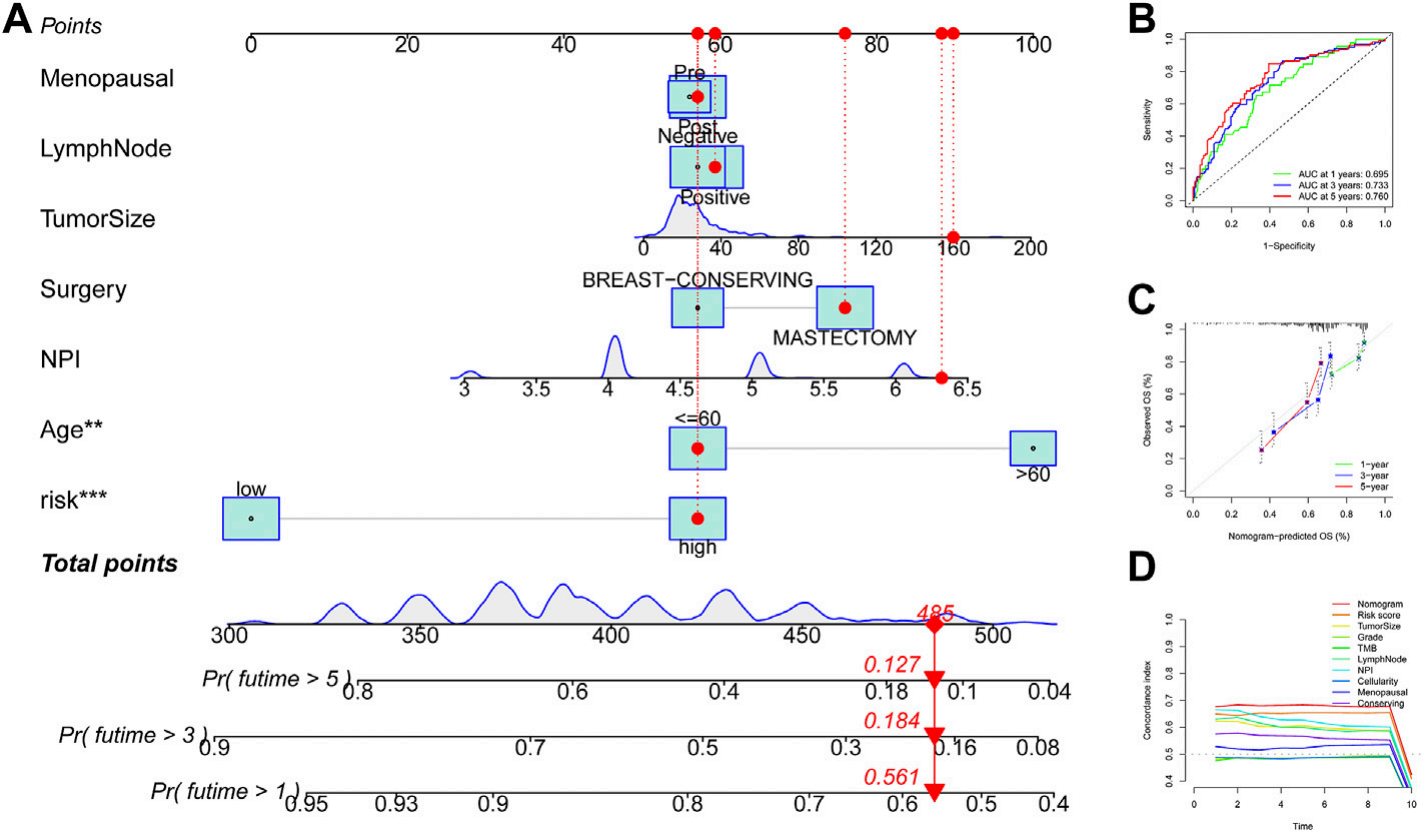
Construction and evaluation of the nomogram model in TNBC. **(A)** Construction of a nomogram model based on the risk score and other clinicopathological factors. **(B)** ROC curve analysis of the nomogram model for predicting 1-, 3-, and 5-year OS in TNBC patients. **(C)** Calibration curve validation of the nomogram model for predicting 1-, 3-, and 5-year OS in TNBC patients. **(D)** C-index of the nomogram model, 11-URG signature-based risk score, and other clinicopathological factors. ROC, receiver operating characteristic; OS, overall survival; and C-index, concordance index.

Univariate Cox regression analysis suggested that the 11-URG signature-based risk score, lymph node status, NPI, menopausal status, and breast conserving surgery were significantly associated with patients’ survival (Figure 8A). We further performed multivariate Cox regression analysis using these factors. The results revealed that the 11-URG signature was the only factor related to the OS of TNBC patients (Figure 8B). Using age, menopausal status, lymph node status, tumor size, surgery, NPI, and risk score, we constructed a prognostic nomogram model to predict the OS of individual TNBC patients (Figure 9A). The AUC of this nomogram for predicting 1-, 3-, and 5-year OS was 0.695, 0.733, and 0.760, respectively (Figure 9B). We applied calibration curves to further assess the predictive effect of the nomogram model on the OS of TNBC patients. As shown in Figure 9C, the nomogram-predicted OS of TNBC patients had good consistency with the actual OS of TNBC patients. The C-index of the nomogram model and 11-URG signature-based risk score were higher than other clinicopathological factors (Figure 9D), suggesting the favorable predictive ability of the nomogram model and 11-URG signature-based risk score.

### Gene set enrichment analysis between high-risk and low-risk group patients

**FIGURE 10:**
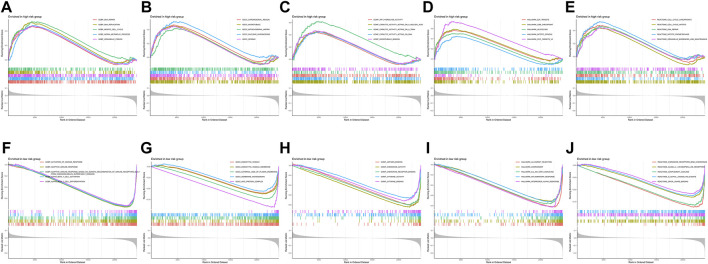
Gene set enrichment analysis between the high-risk and low-risk groups. **(A–E)** Biological process, cellular component, molecular function, hallmark gene set, and reactome pathways enriched in the high-risk group. **(F–J)** Biological process, cellular component, molecular function, hallmark gene set, and reactome pathways enriched in the low-risk group.

As shown in Supplementary Figure S4, high-risk group patients were more sensitive to A-443654, JW-7-52-1, NSC-87877, and PF-4708671 therapy, while low-risk group patients were more sensitive to the remaining chemotherapy drugs such as AZD2281 (olaparib), gefitinib, and nilotinib. The AZD2281 target is at PARP1/2 to influence genome integrity, gefitinib target at the EGFR signaling pathway, and nilotinib target at the ABL signaling pathway. The PI3K/mTOR signaling pathway is the target of A-443654, JW-7-52-1, and PF-4708671, which indicates that high-risk group patients may benefit from therapy targeting at the PI3K/mTOR signaling pathway. To explore the difference in biological characteristics between high-risk and low-risk group patients, we performed GSEA. The GSEA results are presented in Figure 10. The BP of immune response, T-cell activation, and differentiation were enriched in the low-risk group, while the BP of DNA repair, DNA replication, and meiotic cell cycle were enriched in the high-risk group. The CC of genes in the low-risk group was mainly enriched in endocytic vesicles, while the CC of genes in the high-risk group was mainly enriched in chromosomal regions and microtubules. Chemokine and cytokine activities were the enriched MF in the low-risk group; however, ATP hydrolysis activity and catalytic activity were the mainly enriched MF in the high-risk group. Interferon α response, IL6–JAK-STAT3 signaling, complement, and inflammatory response were the mainly enriched hallmark gene sets in the low-risk group, while E2F target, G2M checkpoint, glycolysis, and MYC target were the mainly enriched hallmark gene sets in the high-risk group. As for the reactome pathway, chemokine receptors that bind chemokines and complement cascades were the mainly enriched pathways, while the cell cycle-related pathway and DNA repair were the mainly enriched pathways.

### Immune infiltration and tumor immune microenvironment analyses

**FIGURE 11:**
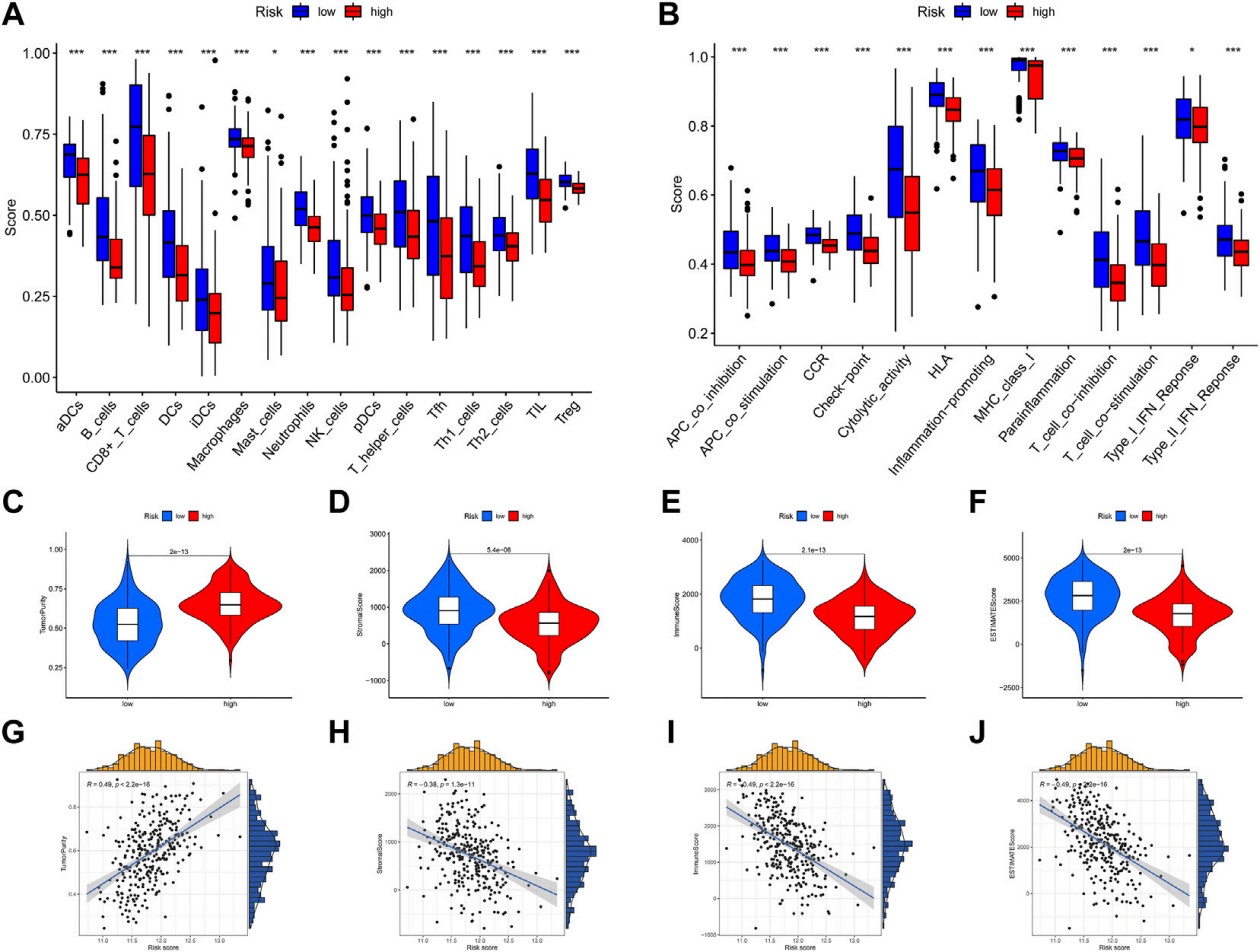
Immune infiltration and tumor immune microenvironment analyses. **(A,B)** ssGSEA scores of 16 immune cells and 13 immune-related functions between the high-risk and low-risk groups. **(C–F)** Violin plot of tumor purity, stromal score, immune score, and ESTIMATE score between the high-risk and low-risk groups. **(G–J)** Scatter plot of the correlation of the risk score with tumor purity, stromal score, immune score, and ESTIMATE score. *p < .05; **p < .01; ***p < .001.

To further identify the potential mechanism of the heterogeneity between high-risk and low-risk group patients, we conducted tumor immune microenvironment and immune infiltration analyses. All of the immune cells and immune-related pathways were higher in the low-risk group than in the high-risk group (Figures 11A, B). Compared with the low-risk group, the high-risk group exhibited higher tumor purity (Figure 11C) and lower stromal score (Figure 11D), immune score (Figure 11E), and ESTIMATE score (Figure 11F). In addition, the risk score was positively correlated with tumor purity (Figure 11G) and negatively correlated with the stromal score (Figure 11H), immune score (Figure 11I), and ESTIMATE score (Figure 11J). As shown in Supplementary Figure S5, the MCP-counter analysis showed that most immune cells (except fibroblasts and neutrophils) are enriched in the low-risk group, which is consistent with ssGSEA. The CIBERSORT analysis (Supplementary Figure S6) showed that B cells, plasma cells, CD4 T cells, and DC cells are enriched in the low-risk group, and M2 macrophage is enriched in the high-risk group. Therefore, both CIBERSORT and MCP-counter analyses are consistent with ssGSEA and ESTIMATE analyses.

## Discussion

In the present study, we identified 17 prognostic URGs and constructed four molecular classifications of TNBC. The immune signatures were significantly different among distinct TNBC clusters. Then, we developed the 11-URG signature with good performance for patients with TNBC. According to the 11-URG signature, TNBC patients can be classified into a high-risk group and a low-risk group with a significantly different OS. The predictive ability of the 11-URG signature was validated in the test set (GSE58812). Univariate and multivariate Cox regression analyses showed that the 11-URG signature was an independent risk factor for TNBC patients. Moreover, we constructed a nomogram comprising the risk score and clinicopathological characteristics with favorable predictive ability. GSEA showed that enriched GO terms, hallmark gene sets, and reactome pathways were evidently different between the high-risk and low-risk groups. In addition, tumor immune microenvironment and immune infiltration analyses also exhibited a significant difference.

TNBC is a heterogeneous cancer. Tailored treatment based on molecular subtypes is meaningful for improving the outcomes of TNBC. In our study, we constructed four clusters of TNBC patients, including 41 patients in cluster 1, 154 patients in cluster 2, 164 patients in cluster 3, and 44 patients in cluster 4. As cluster 2 patients had the worst OS and cluster 4 patients had the best OS among all clusters, we conducted further analysis to reveal the mechanism. GSVA of the pathway showed that glycolysis, cholesterol homeostasis, hypoxia, and DNA repair pathways were the mainly upregulated pathways in cluster 2, while interferon response and inflammatory response were the mainly upregulated pathways in cluster 4. As is known, metabolic reprogramming is one of the hallmarks of cancer. Metabolic reprogramming is used by TNBC to fulfill bioenergetic and biosynthetic demands; maintain the redox balance; and further promote oncogenic signaling, cell proliferation, and metastasis (Wang P et al., 2020). Metabolic reprogramming mainly consists of glycolysis, amino acid metabolism, and lipid metabolism. It has been reported that TNBC cells predominantly use glycolysis for energy production regardless of abundant oxygen availability (Wu et al., 2020). The upregulation of enzymes involved in the glycolytic pathway including hexokinase (Lucantoni et al., 2018), phosphofructokinase (Coelho et al., 2011), pyruvate kinase (Wahdan-Alaswad et al., 2018), and lactate dehydrogenase (McCleland et al., 2012) contributes to the “Warburg effect” in TNBC. The glycolytic phenotype favors TNBC to synchronize with an accelerated rate of proliferation, migration and invasion, and chemotherapy resistance (Arundhathi et al., 2021; Wiggs et al., 2022). Cholesterol, a component of cell membranes, also serves as a precursor for steroid hormones, bile acids, and vitamin D. As a critical molecule for cell growth and function, cholesterol has been recognized as a characteristic of some malignancies (Tosi and Tugnoli, 2005). Statins and hypocholesterolemic drugs that selectively inhibit hydroxymethylglutaryl coenzyme A reductase (HMGCR) also show anticancer activity (Clendening and Penn, 2012). Nevertheless, the function of cholesterol in breast cancer is conflicting (Nelson, 2018; Garcia-Estevez and Moreno-Bueno, 2019). Some researchers found that cholesterol has a protective effect, while other authors concluded that cholesterol is a risk factor, and some found no effect. Importantly, deregulation of cholesterol homeostasis leading to an imbalance of intracellular cholesterol is a crucial regulator for breast cancer (Nazih and Bard, 2020). Hypoxia has long been considered one of the hallmarks of cancer (Gilkes et al., 2014). Several studies (Kapinova et al., 2018; Zheng et al., 2020; Sun et al., 2021; Yang et al., 2021) have systematically analyzed the hypoxia-related gene and constructed prognostic models based on hypoxia-related genes in TNBC. As a result, the difference in enriched pathways between clusters 2 and 4 may be one of the reasons that cluster 4 patients have better OS than cluster 2 patients. We further analyzed the immune microenvironment in four clusters. First, we found that cluster 2 TNBC patients had high tumor purity, a low immune score, and low expression of HLA. In addition, the results of infiltration analysis showed that T cells, CD8 T cells, NK cells, myeloid dendritic cells, cytotoxic lymphocytes, and B lineage were significantly downregulated in cluster 2 and upregulated in cluster 4, while M2 macrophage was significantly upregulated in cluster 2. Finally, immune-related activities including cytolytic activity, inflammation-promoting activity, and the IFN response were also inhibited in cluster 2. Taken together, cluster 2 exhibited the immune desert phenotype, and cluster 4 exhibited the immune-enriched phenotype, which may account for the OS difference between clusters 2 and 4. Our analysis provides new insight into the molecular classification of TNBC patients.

To the best of our knowledge, there are numerous specific gene-based prognostic prediction models for TNBC such as the hypoxia-related gene signature (Zheng et al., 2020), immune-related gene signature (Wang Z et al., 2020; Sun and Zhang, 2022), and autophagy-related gene signature (Yan et al., 2022). However, few have considered the integrated roles of URG set in TNBC. Herein, we developed the 11-URG signature with good performance for patients with TNBC. Of the 11 URGs, HECTD3, PCGF1, RNF123, STC1, GRWD1, USP30, OTUB2, and ATXN3L are the risk factors of TNBC, and BIRC3, STAMBPL1, and PARP11 are the protective factors of TNBC. We further discuss the functions of these URGs. In reviewing the literature, no data were found on the association of breast cancer with PCGF1, RNF123, GRWD1, USP30, ATXN3L, and PARP11. The remaining five URGs were reported to be implicated in breast cancer. HECTD3 is an oncogene and could promote breast cancer cell survival (Li et al., 2013; Jiang et al., 2020), which is in line with the result of our study that HECTD3 is a risk factor for TNBC. Our study supports the evidence that STC1 is a biomarker of breast cancer and promotes tumor growth and metastasis (Chang et al., 2015; Avalle et al., 2022). OTUB2 was reported to promote the progression of gastric cancer and colorectal cancer (Ouyang et al., 2022; Yu et al., 2022). As for breast cancer, a recent study reported that OTUB2 deubiquitinated and activated YAP/TAZ to promote cancer stemness and metastasis (Zhang et al., 2019), which confirms the reliability of our results. Interestingly, BIRC3 can play a tumor-suppressing role or act as an oncogene in different types of cancer (Frazzi, 2021). As for breast cancer, the function of BIRC3 has not yet been fully characterized. Our results corroborate the findings that BIRC3 functions as a tumor suppressor. The study by Liu et al. (2022) found that STAMBPL1 interacts with MKP-1 and stabilizes MKP-1 *via* deubiquitination, further promoting breast cancer cell resistance to cisplatin. Moreover, STAMBPL1 could regulate snail stability by deubiquitination mechanisms in breast cancer (Ambroise et al., 2020). The aforementioned studies showed that STAMBPL1 is a risk factor for breast cancer, which is contrary to our result. Nonetheless, whether STAMBPL1 influences the prognosis of breast cancer patients is unclear and requires further research. Although the 11 URGs have been suggested to be involved in multiple cancers, studies concerning the effects of these URGs on TNBC are lacking. Therefore, the roles of these URGs in TNBC remain unexplored.

According to the 11-URG signature, TNBC patients can be classified into a high-risk group and a low-risk group with significantly different OS. We first analyzed the sensitivity of TNBC patients to chemotherapy drugs. Low-risk group patients are more sensitive to the majority of the chemotherapy drugs than patients in the high-risk group, which may partially explain the preferable OS of low-risk group patients. Then, we conducted GSEA to explore the mechanism. What stands out in the GSEA is the pathway difference between the high-risk and low-risk groups. Cell cycle- and glycolysis-related pathways were the mainly enriched pathways in the high-risk group, while chemokine receptors that bind chemokines, the inflammatory response, and complement cascades were the mainly enriched pathways in the low-risk group. As discussed earlier, the glycolytic phenotype promotes the proliferation, migration, and invasion of TNBC cells. Deregulation of the cell cycle is also a hallmark of cancer that enables limitless cell division and is frequently observed in breast cancer (Thu et al., 2018; Sofi et al., 2022). Therefore, the pathway difference may also explain the prognosis difference between the high-risk and low-risk groups. Furthermore, all of the immune cells and immune-related pathways were higher in the low-risk group than in the high-risk group. The OS difference between the high-risk and low-risk groups could be attributed to the immune infiltration difference between the two groups.

Conventional clinicopathological predictors such as age, gender, and the TNM staging system are insufficient to predict the prognosis of breast patients due to molecule complexity and biological heterogeneity of breast cancer. To provide a quantitative tool for predicting the survival rate of TNBC patients, we constructed a nomogram comprising the 11-URG signature-based risk score and clinicopathological characteristics. The ROC curve and calibration curve suggested that the nomogram is a stable and reliable predictor for OS of TNBC patients.

Admittedly, our study has some limitations because it was based only on high-throughput RNA-sequencing, array profiles, and data analysis. The roles of these prognostic URGs require further *in vitro* and *in vivo* studies because of their strong relevance to the prognosis of TNBC. In conclusion, we identified four novel URGs based on the molecular classification of TNBC and constructed the 11-URG signature with good performance for patients with TNBC. Our analysis suggests that URGs play important roles in TNBC and are potential prognostic biomarkers and therapeutic targets.

## Data Availability

The original contributions presented in the study are included in the article/Supplementary Material, further inquiries can be directed to the corresponding author.

## Supplementary material

SUPPLEMENTARY FIGURE S1 Heatmap of ubiquitination-related gene expression among four clusters of TNBC.

SUPPLEMENTARY FIGURE S2 Immune microenvironment analysis of TNBC clusters. Violin plot of **(A)** ESTIMATE score, **(B)** immune score, **(C)** stromal score, and **(D)** tumor purity.

SUPPLEMENTARY FIGURE S3 Immune infiltration analysis of TNBC clusters. Violin plot of immune cell infiltration and immune-related activities among four clusters.

SUPPLEMENTARY FIGURE S4 Sensitivity of TNBC patients to chemotherapy drugs between the high-risk and low-risk groups.

SUPPLEMENTARY FIGURE S5 MCP-counter analysis of immune cell infiltration between the high-risk and low-risk groups.

SUPPLEMENTARY FIGURE S6 CIBESORT analysis of immune cell infiltration between the high-risk and low-risk groups.
