# Supplementary figures and images for: Identification of ubiquitination-related gene classification and a novel ubiquitination-related gene signature for patients with triple-negative breast cancer
Source: Front Genet. 2023 Jan 6;13:932027. doi: 10.3389/fgene.2022.932027 (PMC9853012; doi:10.3389/fgene.2022.932027)

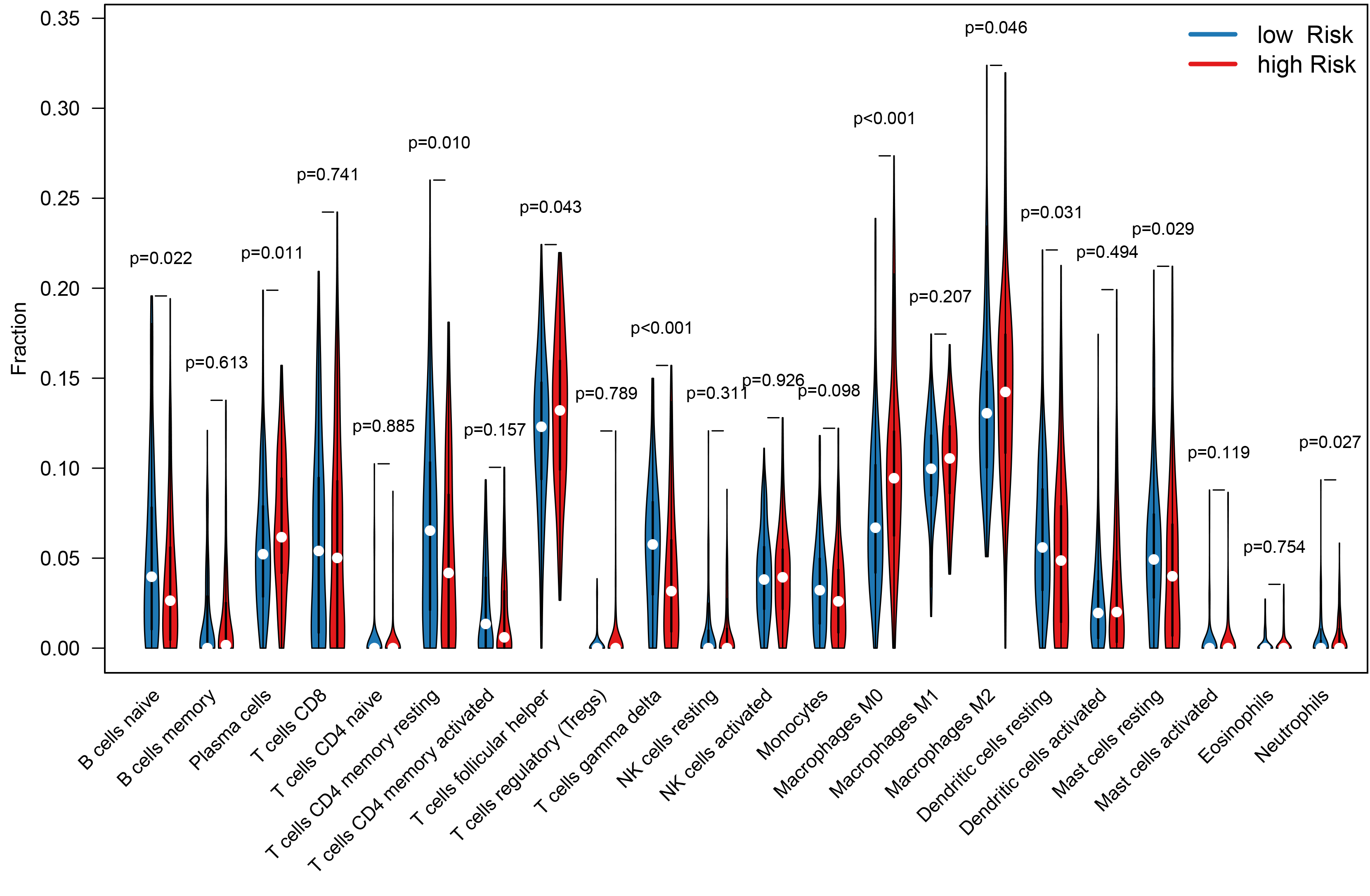

Supplement: Supplementary file 2 [file Image6.TIF]

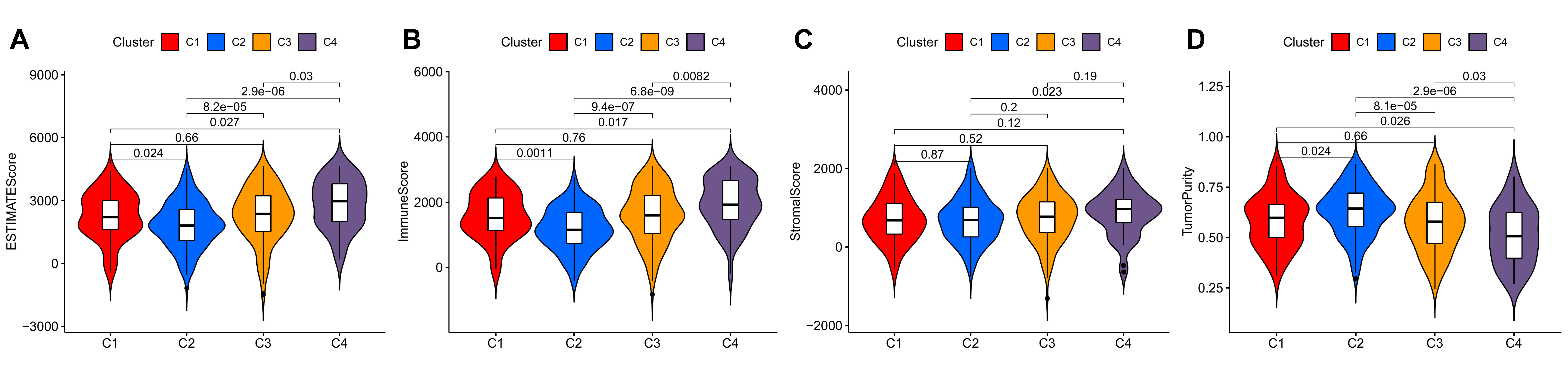

Supplement: Supplementary file 4 [file Image2.JPEG]

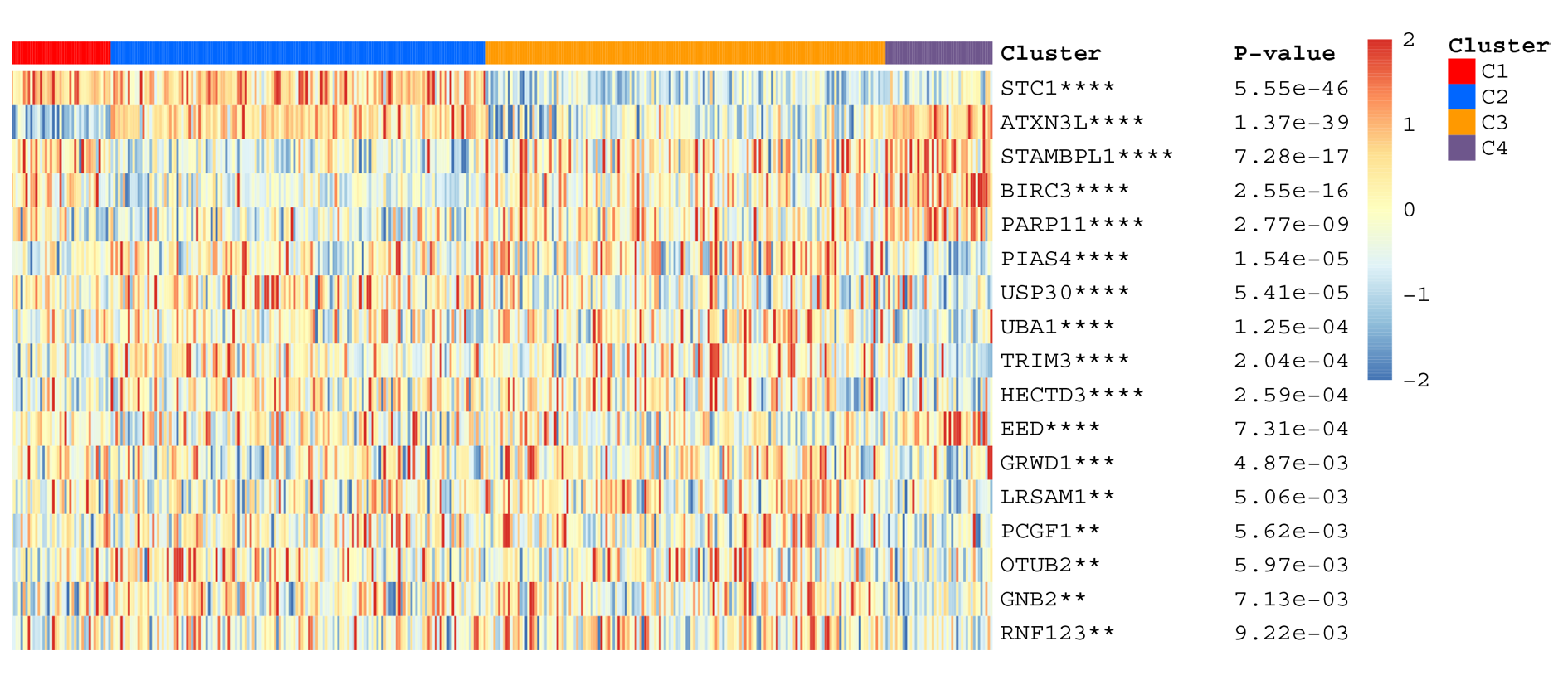

Supplement: Supplementary file 5 [file Image1.TIF]

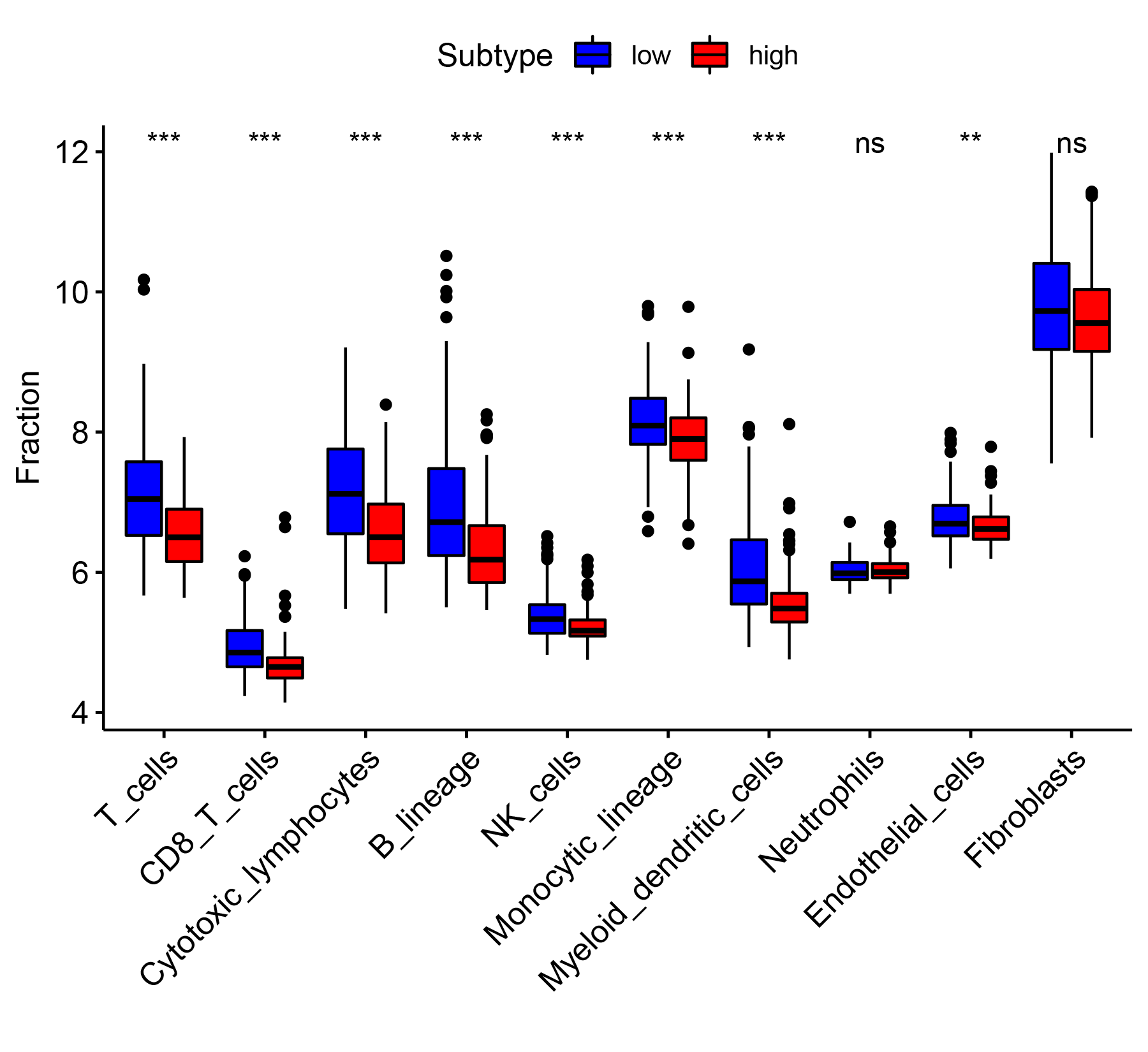

Supplement: Supplementary file 6 [file Image5.TIF]
